# Association of the SNP in *akirin 2* Gene With Growth and Carcass Traits in Zavot Cattle

**DOI:** 10.1002/vms3.70161

**Published:** 2025-01-24

**Authors:** Osman Tufan Ertan, Yunus Arzik, Fadime Daldaban, Korhan Arslan, Bilal Akyuz

**Affiliations:** ^1^ Erciyes University, Institute of Health Sciences, Department of Veterinary Animal Science Kayseri Turkey; ^2^ Faculty of Veterinary Medicine Aksaray University Aksaray Turkey; ^3^ Faculty of Veterinary Medicine, Erciyes University Kayseri Turkey

**Keywords:** *AKIRIN2*, average daily live weight gain, cattle, meat yield, percentage

## Abstract

Understanding the genetic factors that influence meat yield is crucial due to the economic importance of average daily live weight gain (ADWG) in livestock. This study investigates the relationship between the c.*188G>A SNP in the 3′‐UTR region of the *akirin 2* gene and growth traits in Zavot cattle, focusing on the gene's role in muscle development. Genotyping of the c.*188G>A SNP was conducted using polymerase chain reaction–restriction fragment length polymorphism analysis, revealing frequencies of 0.09 for AA, 0.75 for AG and 0.16 for GG genotypes, respectively. Our findings demonstrate a significant association between this SNP and ADWG, as well as percentage. These results suggest that the c.*188G>A SNP within *akirin 2* could serve as a valuable DNA marker for predicting ADWG and percentage traits in Zavot cattle.

## Graphical abstract

This study explores the association between the *c.188G>A SNP in the **3′‐UTR region of the *akirin 2* gene** and key growth traits in **Zavot cattle**, specifically focusing on its impact on **average daily live weight gain (ADWG)**. Using **polymerase chain reaction–restriction fragment length polymorphism (PCR‐RFLP) analysis**, we identified genotype frequencies of **0.09 (AA)**, **0.75 (AG)** and **0.16 (GG)**. The *c.188G>A SNP showed a significant correlation with **ADWG and percentage traits**, suggesting that it could serve as a valuable **DNA marker** for improving growth performance in Zavot cattle.

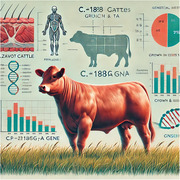

## 1 Introduction

Considering the rapid increase in global population growth and the limited production of basic animal‐based nutrients, it becomes necessary for the livestock sector to adopt more efficient and sustainable production models. This pressing need highlights a key challenge within the current worldwide agricultural and livestock production systems. Given the limited available resources, the struggle to sustainably nourish an expanding population is a requirement in the foreseeable future (Eggen 2012). Furthermore, in a balanced and healthy diet, each individual must consume at least 50% of animal‐origin food (Lorcu and Bolat 2012). The total amount of protein consumed per person per day in Türkiye is 111.1 g on average (Fao 2018), and most of this protein source is met from cattle. Unfortunately, in such countries as Türkiye, the capacity of grazing areas is limited for pasture‐based livestock activities. Therefore, livestock keepers mainly engage in intensive production and prefer dual‐purpose (i.e., dairy and meat) breeds.

In Türkiye, approximately 1.2 million tonnes of red meat are produced yearly, with beef and dairy cattle contributing significantly to this production (TUIK, 2020). However, akin to global trends, the demand surge for food in tandem with population growth has rendered indigenous breeds less desirable due to their lower meat and milk yields, leading to a heightened risk of their extinction (Čítek et al. 2006). Hence, there is a compelling need to increase the performance and product quality of native domestic breeds to ensure their preservation in a sustainable manner (Coşkun and Akyüz 2016).

Zavot, a native cattle breed originating from the Kars and Ardahan provinces in Northeastern Türkiye, stands as a valuable genetic asset within both the Turkish and global cattle breeds. Despite its superiority over other native breeds in terms of productivity, Zavot remains the least recognized and endangered among the native cattle breeds. The breed was developed through the crossbreeding of Simmental and Swiss Brown cattle, introduced to the region from Russia around 150 years ago, with the Eastern Anatolian Red cattle, the indigenous breed of the area (Sariözkan, Akcay, and Bayram 2013; Boğa Kuru, Kırmızıbayrak, and Özşensoy 2022). Zavot cattle, recognized as a dual‐purpose breed, are raised for both milk and meat yield (Akçay, Akyüz, and Bayram 2015).

This increase in demand for animal‐derived food has increased the tendency toward rapid, sustainable and efficient animal production methodologies (Ertan and Akyüz 2021). Traditionally, livestock selection practices relied on classical selection methods (Şahin, Öner, and Elmacı 2013). However, the protracted generation interval, particularly in cattle, renders classical methods such as progeny testing both costly and challenging (Schaeffer 2006; Thornton 2010; Bouquet and Juga 2013). Conversely, advancements in genomic studies in farm animals, unveiling crucial genetic information like reference genomes, have showcased the potential for swifter genetic enhancements in production traits at a reduced long‐term cost compared to classical methods. In addition, marker‐assisted selection (MAS) methods, leveraging genomic variations such as quantitative trait loci (QTL), single nucleotide polymorphisms (SNP), and copy number variation (CNV), particularly in cattle, represent an important paradigm change in breeding approaches (Čítek et al. 2006; Özşensoy and Kurar 2013).

Within farm animals such as cattle and sheep, economically significant traits encompass growth, carcass characteristics and meat quality traits (Warner et al. 2010; Kizilaslan et al. 2022; Yilmaz et al. 2022). Notably, genetic studies targeting meat production and quality traits in cattle have surged, demonstrating their substantial impact on beef cattle breeding (Elmacı and Öner 2007). Studies focused on the genetics of meat production and quality traits have identified numerous genes associated with these attributes, and some of them have already been used in cattle breeding (Casas et al. 2005; X. Li et al. 2013; Y. Li, Cheng, Zhao, et al. 2020; Cheng et al. 2022; Yilmaz et al. 2022). Specifically, the *akirin 2* gene, located on BTA9, has emerged as a critical gene implicated in immune response, skeletal myogenesis, muscle development and carcinogenesis (Zeng et al. 2022; Yang et al. 2024). Previous research underscores its association with the marbling score in cattle, highlighting its potential significance in marker‐assisted selection endeavours (Goto et al. 2008; Sasaki et al. 2009; Watanabe et al. 2012; Kim et al. 2013; Sukegawa et al. 2014; Ma et al. 2015). Moreover, the c.*188G>A SNP within the 3′‐untranslated region (UTR) of *akirin 2* has previously been linked to the marbling score in Japanese Black cattle (Sasaki et al. 2009).

However, the association between the c.*188G>A SNP of *akirin 2* and economically important growth traits within the native Zavot cattle breed remains unexplored. Thus, this study aims to elucidate the relationship between the c.*188G>A SNP on *akirin 2* and the economically significant growth and carcass traits, including live weight, chest girth, average daily weight gain (ADWG) at the ages of 12 and 18 months, slaughter weight, hot carcass weight, carcass yield, liver weight, dress weight, cold carcass weight and cooling loss in the Zavot breed.

## 2 Materials and Methods

### 2.1 Study Population and Phenotypic Data

A total of 44 male cattle from the Zavot breed were included in the study. Initially, the cattle were grazed on pasture until reaching one year of age, after which they underwent intensive fattening. Throughout the six‐month intensive fattening period, the animals were provided with ad libitum access to a ration consisting of corn silage, hay, and concentrated feed. Upon completion of the fattening process, the cattle were sent for slaughter. Detailed records were maintained, including basic information such as birthdates, fattening durations, and slaughter ages. In addition, growth traits such as live weight, chest girth and ADWG at the ages of 12 and 18 months, as well as live weights at slaughter, were documented. All animals were slaughtered following a 24‐h fasting period, and the carcasses were chilled for 24 h at 4°C. After culling the animal, carcass traits, encompassing hot carcass weight, carcass yield, liver weight, dress weight and both hot and cold carcass weights, along with cooling loss, were also recorded. These records were compiled to provide a comprehensive profile of the growth and carcass characteristics of the animals under study. The descriptive statistics of the growth and carcass traits are presented in Table 1.

**TABLE 1:** The descriptive statistics of growth and carcass traits.

Traits	Parameters					
	*N*	Mean	SD^a^	Minimum	Maximum	CV%^b^
BW12 (kg)	44	277.96	36.12	213.29	360.29	0.12
CG12 (cm)	44	154.83	7.38	140.00	168.00	0.04
ADWG12 (kg)	44	1.16	0.33	0.50	1.86	0.29
BW18 (kg)	44	409.93	47.61	331.37	519.17	0.11
CG18 (cm)	44	179.61	7.27	164.00	195.00	0.04
ADWG18 (kg)	44	1.35	0.32	0.73	2.06	0.24
SW (kg)	44	518.40	53.27	405.38	666.09	0.10
CGSL (cm)	44	190.79	6.51	178.00	208.00	0.03
HC (kg)	44	300.89	32.20	227.00	366.80	0.10
Percentage (%)	44	58.02	0.01	0.54	0.62	0.03
Liver (kg)	44	6.22	0.80	4.86	8.16	0.13
DW (kg)	44	44.90	5.99	32.14	61.03	0.13
CC (kg)	44	296.26	31.77	223.60	363.20	0.10
CL (kg)	44	4.62	1.76	1.80	8.80	0.38

^a^ Standard deviation.

^b^ Coefficient of variation.

### 2.2 DNA Extraction and Genotyping

Genomic DNA was extracted using the phenol/chloroform/isoamyl alcohol (25:24:1) method from whole blood (Sambrook et al. 1989). For genotyping of the c.*188G>A SNP in the *akirin 2* gene of the examined samples, a primer set was utilized, comprising forward: 5ʹ‐TCT TAG GCA GCA ACC GGA TT‐3ʹ and reverse: 5ʹ‐GAA GGG CAT GTT CTT AGA ATA CCA G‐3ʹ (GenBank accession number: NC_007329) (Kim et al. 2013).

The PCR mixture was prepared by adding 1.5 µL of DNA (100 ng/µL), 0.1 µL of Taq polymerase (5 U/µL), 50 µM of dNTP and 0.2 µM of each primer, and then supplemented with ddH2O to achieve a total reaction volume of 25 µL. The tubes containing the prepared mixture were held at 94°C for 4 min in a thermal cycler, followed by 35 cycles as follows: 30 s at 94°C, 30 s at 56°C and 30 s at 72°C. After the final cycle, the PCR process was concluded by holding at 72°C for 4 min. Subsequently, the obtained PCR products were digested for each sample using 10 U of *Fok*I (Thermo Scientific) restriction endonuclease enzyme.

### 2.3 Statistical Analysis

The genotypic structures and allele frequencies of individuals were determined by gene counting. Allele and genotype frequencies of samples from the examined breeds were calculated, and Hardy‐Weinberg equilibrium (HWE) was assessed. The HWE Chi‐square test was performed using the R statistical program.

A general linear model (GLM) was used for the association analysis between SNPs and selected body traits. The statistical linear model for this analysis is below:

$$Y_{ij}=\mu +g_{i}+a_{j}+e_{ij}$$

where *Y* is the observation of each trait per individual, *μ* is the overall population mean per trait, *g_i_* is the fixed effect associated with genotype with three levels, *a_j_* is the fixed effect of age and *e* is the standard error.

Tukey's HSD post hoc test was implemented to identify specific differences among genotype groups. R statistical programming, along with various basic packages, was utilized for both data management and advanced statistical analyses.

## 3 Result and Discussion

### 3.1 Genetic Diversity Analysis of the SNP

**FIGURE 1:**
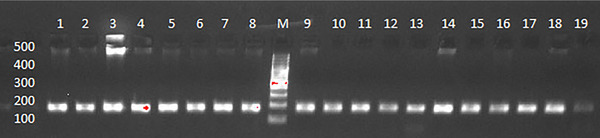
PCR products (a: 170 bp band; M: 100 bp DNA ladder).

The 170‐base pair (bp) products obtained at the end of PCR were analysed with 3% success by agarose gel electrophoresis visualized (Figure 1).

**FIGURE 2:**
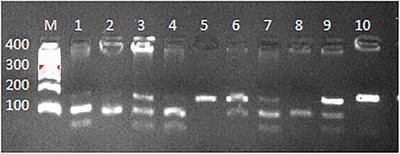
*FoK*I enzyme digestion products of different Akirin 2 genotypes; 5 and 10 individuals of the AA genotype; 1,3,6,7,9 and individuals of the AG genotype; 8 and 2 individuals of the GG genotype; M: 100 bp DNA ladder.

It was expected to see a single band of 170 bp in individuals with the AA genotype, three bands of 170, 105 and 65 bp in heterozygous individuals with the AG genotype, and two bands of 105 and 65 bp in individuals with the GG genotype at the end of cutting the PCR 170 bp products obtained with the *FoK*I restriction endonuclease. However, the smallest band with a band size of 65 bp could not be seen in the 3% agarose gel electrophoresis in which the cut products were run. However, the appearance of 170 and 105 bp bands together or separately was found to be sufficient to determine the genotypes of the individuals, and it was not necessary to increase the gel density to visualize the 65 bp band (Figure 2). The agarose electrophoresis patterns of the c.*188G>A SNP in the *AKIRIN2* gene are illustrated in Figure 2.

*FoK*I endonuclease enzyme of the PCR products obtained c.*188G>A analysis based on the gel images obtained from genotyping as a result of cuttings revealed three distinct genotypes.

The most prevalent genotype observed was AG (75%), while the GG and AA genotypes were relatively less represented within the studied population at 16% and 9%, respectively. Table 2 presents the genotype and allele frequencies within the studied population. The chi‐square value in Table 1 shows that the population is not at HWE.

**TABLE 2:** Genotype and allele frequencies of the c.*188G>A SNP in the *AKIRIN2* gene in the population.

	Genotype frequency (%)			Allele frequency		
	GG	GA	AA	G	A	Chi‐square analyses (HWE)
**Observed**	7 (0.16)	33 (0.75)	4 (0.09)	0.54	0.46	*χ* ^2^ = 11.12
**Expected**	12.3	22	9.7			*p* < 0.05 with df = 1

In a previous study by Coşkun and Akyüz (2016), which investigated this SNP, the GG genotype frequency was found to be high in the East Anatolian Red (EAR), Simmental and Brown Swiss breeds, contributing to the formation of the Zavot breed (0.85, 0.54 and 0.55, respectively) (Coşkun and Akyüz 2016). Specifically, the GG genotype frequency was observed to be higher in the EAR breed (0.85) compared to other breeds. In the current study, the most prevalent genotype was GA (0.75), whereas the AA genotype was found to be the least common in the Zavot breed. This observation aligns with the findings of Coşkun and Akyüz (2016), where the EAR, Simmental and Brown Swiss AA genotypes, contributors to the Zavot breed, were reported to be the least common. It is speculated that the non‐equilibrium in Hardy‐Weinberg balance for the c.*188G>A SNP in the *AKIRIN2* gene among the examined Zavot breed cattle might be attributed to the higher prevalence of the GA genotype compared to homozygous genotypes.

A review of the literature reveals limited research on the c.*188G>A polymorphism in the *akirin 2* gene in cattle. Notably, in a study involving Japanese Black cattle, the GA genotype was found to be predominant (0.48), whereas AA and GG genotype frequencies were equal (0.26) (Sasaki et al. 2009). Nevertheless, consistent with our study, Sukegawa et al. (2014) reported a higher frequency of the GA genotype (0.53) and G allele (0.57) in Japanese Black cattle raised for fattening. In contrast, an examination of Korean native cattle, without planned selection, revealed a notably low frequency of the AA genotype (0.07), similar to our study (Kim et al. 2013).

In population genetics, the frequency of heterozygotes serves as a pivotal metric delineating the genetic diversity within a given population. Elevated frequencies of heterozygotes within a locus indicate augmented genetic variability or diversity at that specific genetic marker within the population (Charlesworth 2013). Several reasons elucidate the presence of higher heterozygosity at a locus or quantitative trait locus (QTL), including genetic diversity, balanced selection, outbreeding and recent population expansion (Magurran 1998; Casillas and Barbadilla 2017). The amplification of heterozygosity often signifies a richer assortment of diverse alleles present at the locus in the population. This diversity substantiates the population's potential to adapt to dynamic environmental shifts and resistance against various diseases. In addition, selective pressure, such as natural selection, occasionally favours the maintenance of genetic diversity (Kantanen et al. 2000). For example, heterozygote advantage, wherein individuals harbouring two distinct alleles at a locus enjoy a selective edge over homozygotes, may drive the escalation of heterozygosity. Furthermore, populations engaging in recurrent outbreeding practices, characterized by mating with individuals from divergent populations, typically exhibit heightened levels of heterozygosity. The propensity for disparate alleles to manifest at a locus escalates owing to the enhanced genetic diversity introduced through outbreeding events (Notter 1999). Finally, populations emanating from recent expansions originating from smaller founding populations may manifest increased heterozygosity due to the genetic variety inherent within the initial, more confined group.

All in all, when considering the limited population size of the Zavot breed used in the study in Türkiye, along with uncontrolled crossbreeding practices with exotic breeds to increase productivity in native breeds and recognizing this breed's high adaptation ability to harsh conditions, the high heterozygosity observed on the c.*188G>A SNP in the study population can be explained by the scenarios mentioned above.

### 3.2 Association Analysis

The effects of the c.*188G>A SNP of *akirin 2* on six growth and six carcass traits was analysed in 44 male Zavot cattle (Table 3). GLM was used to estimate the associations between genotypes and traits. Three different genotype groups were included as factors, and the age of the animal was included as a fixed effect in the model. According to the association analysis, it was found that animals with the AA genotype exhibited statistically higher daily weight gain at the age of 12 months compared to the other genotypes (*p* < 0.05) among the growth traits. Similarly, in carcass traits, the percentage representing the live weight to hot carcass weight ratio was found to be statistically higher in animals with the AA genotype compared to the other genotypes (*p* < 0.05). For other traits, no statistically significant association was found among the genotype groups (Table 3).

**TABLE 3:** Association of the c.*188G>A SNP with growth and carcass traits in Zavot cattle.

Genotype	N	Growth traits (LSM ± SE)						
		BW12 (kg)	CG12 (cm)	ADWG12 (kg)	BW18 (kg)	CG18 (cm)	ADWG18 (kg)	CGSL (cm)
**AA**	4	279 ± 18^a^	152 ± 03^a^	**1.46 ± 0.16^b^**	409 ± 24^a^	180 ± 04^a^	1.41 ± 0.16^a^	191 ± 03^a^
**GA**	33	282 ± 06^a^	156 ± 01^a^	1.10 ± 0.05^a^	415 ± 08^a^	180 ± 01^a^	1.38 ± 0.05^a^	191 ± 01^a^
**GG**	7	261 ± 13^a^	150 ± 02^a^	1.29 ± 0.12^a^	386 ± 18^a^	176 ± 03^a^	1.20 ± 0.12^a^	188 ± 02^a^

		Carcass traits (LSM ± SE)						
		SW (kg)	HC (kg)	Percentage (%)	Liver (kg)	DW (kg)	CC (kg)	CL (kg)
**AA**	4	509 ± 28^a^	302 ± 16^a^	**59.50 ± 0.00^b^**	6.21 ± 0.4^a^	45.30 ± 03^a^	298 ± 16^a^	3.90 ± 0.85^a^
**GA**	33	521 ± 10^a^	301 ± 05^a^	57.80 ± 0.00^a^	6.22 ± 0.1^a^	44.80 ± 01^a^	296 ± 05^a^	4.95 ± 0.30^a^
**GG**	7	514 ± 21^a^	299 ± 12^a^	58.30 ± 0.00^a^	6.25 ± 0.3^a^	45.00 ± 02^a^	296 ± 12^a^	3.51 ± 0.64^a^

*Note*: Values with different letters are significantly different at *p* < 0.05 (a, b) and *p* < 0.05 according to the Tukey HSD test. Values are shown as the least square means ± standard errors.

The bold values indicate results that are statistically significant at *p* < 0.05.

Abbreviations: BW12, CG12 and ADWG12, body weight, chest girth and average daily weight gain at the age of 12 months; BW18, CG18 and ADWG18, body weight chest girth and average daily weight gain at the age of 18 months; CC, cold carcass weight; CGSL, chest girth at slaughter; CL, cooling loss; DW, dress weight; HC, hot carcass weight; SW, slaughter weight.

The c.*188G>A SNP of the *akirin 2* gene demonstrated associations with marbling scores in Japanese Black and Korean native cattle, and this SNP has been reported to be a positional functional candidate SNP between marbling scores and meat quality in cattle (Sasaki et al. 2009; Watanabe et al. 2012; Kim et al. 2013). According to previous studies, *akirin 2* is positioned within the QTL related to carcass and growth traits in Japanese Black, Angus and several crossbred populations (Takasuga et al. 2007; McClure et al. 2010; Mateescu, Garrick, and Reecy 2017). Additionally, *akirin 2* is suggested to play roles in immune response, skeletal myogenesis, muscle development, and carcinogenesis based on previous studies in various species (Goto et al. 2008; Galindo et al. 2009; Zeng et al. 2022). However, there are limited studies investigating the relationship between this SNP and growth and meat yield traits. In one of these studies, Kim et al. (2013) reported that in Korean native cattle, cattle with the AA genotype in terms of c.*188G>A SNP of *akirin 2* had larger musculus longissimus dorsi areas and higher carcass weights than those with GG and AG genotypes. In another study, it was reported that cattle with the GG genotype in Chinese native cattle due to c.*188G>A SNP of *akirin 2* had lower body length, and the SNP was also associated with chest depth and chest circumference, and it was suggested that the SNP could be used as a marker for growth characteristics in Chinese Qinchuan cattle (Y. Li, Cheng, Yamada, et al. 2020). Cheng et al. (2022), in their study on six domestic cattle breeds raised in China, c.*188 SNP>A in the *akirin 2* gene was found to be associated with body sizes; it has been reported that individuals with the GG genotype are lower in terms of body length, hip height, chest depth, and chest circumference.

Our study is one of the first studies to investigate the relationship between c.*188G>A SNP of *akirin 2* and live weight gain in cattle. In our study, ADWG in cattle with the AA genotype was found to be higher than those with other genotypes. ADWG is an important component of the profitability of farm animals raised for meat yield (Nielsen et al. 2013). Cattle with a faster ADWG consume less feed during fattening and reach slaughter weight in a shorter time. This contributes to the profitability of livestock enterprises (MacNeil et al. 2021). Therefore, average daily live weight gain is an important selection criterion in beef cattle breeding programs (Santana et al. 2014). For this reason, selection studies in terms of ADWG will contribute to the profitability of enterprises. As a result of this study, it was thought that the c.*188G>A SNP of *akirin 2* could be used to improve the ADWG trait in beef cattle breeding (Bouquet and Juga 2013; Kantanen et al. 2000).

In all animals raised for meat yield, ADWG and percentage are important factors for productivity and thus profitability. ADWG, which is one of the most important factors affecting the fattening performance of farm animals, affects the feed conversion ratio. One of the most important inputs in cattle breeding is feed consumption. High daily live weight gain affects the feed conversion rate, which reduces the feed cost of the enterprise. In addition, high daily live weight gains enable livestock to reach slaughter weight in a short time, increasing the profitability of the enterprise. In this study, the animals with AA genotypes were observed to have high ADWG (Table 3). For this reason, it was thought that selection for the AA genotype could contribute to increasing the ADWG of the breed.

There is a positive relationship between carcass weight and percentage. Therefore, a high percentage will increase the amount of meat and valuable meat obtained from the slaughtered animals. The profitability of breeders will increase as valuable meat will be sold at higher prices (Sariözkan, Akcay, and Bayram 2013). For this reason, raising a high percentage of animals in fattening enterprises is important for profitability. In the study, it was observed that the percentage of animals with AA genotypes was higher than those with other genotypes (Table 3). Although the live weights of animals with the GG genotype were high at slaughter, their hot carcass weights were observed to be low (Table 3), and it was thought that this may be due to abdominal fat in the animals with the GG genotype.

The beef industry in Türkiye has the rapidly expanding potential to meet the high demand for meat due to its large population. While numerous molecular markers have been identified for breeding purposes, further research is required to discover more effective molecular markers for enhancing cattle breeds. In our current research, the association analysis revealed the impact of the c.*188>A SNP in *akirin 2* on growth and carcass traits, specifically on ADWG and carcass yield, respectively, in Zavot cattle. These findings highlight the potential role of the *akirin 2* gene in cattle breeding aimed at meat production. However, our study's limitation lies in its small sample size. To comprehensively unravel the genetic mechanisms related to the *akirin 2* gene in Zavot and other indigenous cattle breeds in Türkiye, future research requires more sophisticated and well‐structured studies with larger sample sizes representing the actual population.

## 4 Conclusion

The c.*188G>A SNP in the *akirin 2* gene shows promising potential as a marker for growth and carcass traits in Zavot cattle. This study reveals a significant association between the AA genotype and higher ADWG and carcass yield, underscoring the gene's relevance to livestock productivity. The AA genotype demonstrated superior growth performance, making it a viable target for selection in breeding programs aimed at improving meat yield efficiency. However, the small sample size limits the generalizability of the results. Further research is recommended, utilizing larger populations and more advanced phenotypic measures like ultrasound‐based muscle assessments, to fully understand the genetic and biological mechanisms at play. A broader investigation into the gene's role across diverse cattle breeds would help validate its utility in the genetic improvement of livestock, potentially making it a key tool in optimizing meat production efficiency in the Turkish beef industry.

## Author Contributions

**Osman Tufan Ertan**: conceptualization, visualization, writing–original draft, writing–review and editing, methodology. **Yunus Arzik**: conceptualization, formal analysis, writing–original draft, writing–review and editing, methodology, software. **Fadime Daldaban**: conceptualization, formal analysis, investigation. **Korhan Arslan**: conceptualization, data curation, methodology, investigation. **Bilal Akyuz**: conceptualization, data curation, writing–review and editing, writing–original draft, supervision, validation, funding acquisition, project administration.

## Ethical Statement

The study measurements were conducted under the supervision of Erciyes University Local Ethics Committee for Animal Experiments (HADYEK). Methods were carried out following the ARRIVE Guidelines 2.0.

## Conflicts of Interest

The authors declare no conflicts of interest.

## Data Availability

The datasets generated during and/or analysed during the current study are available from the corresponding author upon reasonable request.
